# Effects of Growth Hormone Treatment and Rehabilitation in Incomplete Chronic Traumatic Spinal Cord Injury: Insight from Proteome Analysis

**DOI:** 10.3390/jpm10040183

**Published:** 2020-10-21

**Authors:** Tatiana Martin-Rojas, Tamara Sastre-Oliva, Ana Esclarín-Ruz, Felix Gil-Dones, Laura Mourino-Alvarez, Nerea Corbacho-Alonso, Rafael Moreno-Luna, German Hernandez-Fernandez, Juan Antonio Lopez, Antonio Oliviero, María G. Barderas

**Affiliations:** 1 Department of Vascular Physiopathology, Hospital Nacional de Paraplejicos (HNP), SESCAM, 45071 Toledo, Spain; tatiana_martin_rojas@hotmail.com (T.M.-R.); tsastre@sescam.jccm.es (T.S.-O.); gildones@yahoo.es (F.G.-D.); lmourino@sescam.jccm.es (L.M.-A.); ncorbacho@sescam.jccm.es (N.C.-A.); rmluna@sescam.jccm.es (R.M.-L.); ghernandezf@externas.sescam.jccm.es (G.H.-F.); 2 Department of Physical Medicine and Rehabilitation, Hospital Nacional de Parapléjicos, SESCAM, 45071 Toledo, Spain; anae@sescam.jccm.es; 3 Department of Genetic, Facultad de Ciencias Biológicas, UCM, 28040 Madrid, Spain; 4 Department of Neuroinflammation, Hospital Nacional de Paraplejicos (HNP), SESCAM, 45004 Toledo, Spain; 5 Unidad de Proteomica CNIC, 28029 Madrid, Spain; jalopez@cnic.es; 6 FENNSI Group, Hospital Nacional de Parapléjicos, SESCAM, 45071 Toledo, Spain

**Keywords:** spinal cord injury, somatropin, therapy, proteomic

## Abstract

Despite promising advances in the medical management of spinal cord injury (SCI), there is still no available effective therapy to repair the neurological damage in patients who experience this life-transforming condition. Recently, we performed a phase II/III placebo-controlled randomized trial of safety and efficacy of growth hormone (GH) treatment in incomplete chronic traumatic spinal cord injury. The main findings were that the combined treatment of GH plus rehabilitation treatment is feasible and safe, and that GH but not placebo slightly improves the SCI individual motor score. Moreover, we found that an intensive and long-lasting rehabilitation program per se increases the functional outcome of SCI individuals. To understand the possible mechanisms of the improvement due to GH treatment (motor score) and due to rehabilitation (functional outcome), we used a proteomic approach. Here, we used a multiple proteomic strategy to search for recovery biomarkers in blood plasma with the potential to predict response to somatropin treatment and to delayed intensive rehabilitation. Forty-six patients were recruited and followed for a minimum period of 1 year. Patients were classified into two groups based on their treatment: recombinant somatropin (0.4 mg) or placebo. Both groups received rehabilitation treatment. Our strategy allowed us to perform one of the deepest plasma proteomic analyses thus far, which revealed two proteomic signatures with predictive value: (i) response to recombinant somatropin treatment and (ii) response to rehabilitation. The proteins implicated in these signatures are related to homeostasis, inflammation, and coagulation functions. These findings open novel possibilities to assess and therapeutically manage patients with SCI, which could have a positive impact on their clinical response.

## 1. Introduction

The annual incidence of traumatic spinal cord injury (SCI) in Spain is estimated at 1000 cases, with the vast majority caused by traffic accidents (48%), followed by falls (21%) and sport injuries (14.6%) [1]. In addition, there are also many other cases of non-traumatic SCI of other origins, such as tumors, infectious and vascular diseases, and multiple sclerosis, among others [2]. The most common medical classification to characterize SCI is the International Standards for Neurological Classification of Spinal Cord Injury (ISNCSCI) to classify subjects using the ASIA Impairment Scale (AIS) and the neurological level of SCI (the more caudal neurological level with normal neurological function). ISNCSCI categorizes injuries by severity into complete (patient without sacral preservation; defined as ASIA A), and incomplete (patients with sensorimotor sacral preservation) injury, which is further subdivided into different degrees of involvement (B, C, D and E) [2]. Presently, there is no treatment available for SCI once it is established, and rehabilitation is the only therapy for functional improvement. The life-changing consequences, along with the high social cost and the lack of treatment options make the study of SCI pathophysiology an important scientific and medical objective [3].

The incidence of traumatic SCI is higher in younger persons between 16 and 30 years (and more often in males), and the prevalence is higher in the 40–49 years age group, with chronic lesions of more than 15–20 years (www.christopherreeve.org). Patients with traumatic SCI can show an improvement in motor and sensory functions during the first year, but there is usually little progress after this period [4,5,6]. Accordingly, expert guidelines [6] consider that the clinical condition of patients is unlikely to change after 12–18 months without the aid of rehabilitation, and clinical trials should be performed from this time onwards [5,6].

Growth hormone (GH) is a polypeptide hormone secreted by the anterior pituitary gland that stimulates growth, cell reproduction, and cell regeneration in humans and other animals [7,8]. GH also stimulates the production of insulin growth factor-1 (IGF-1) and increases the concentration of glucose and free fatty acids [9,10,11]. GH is commonly used by physicians as a replacement therapy to treat children with hormone deficiency and growth retardation, and it is now manufactured by recombinant DNA technology-producing somatotropin that has an identical amino acid sequence to that of the isolated human pituitary hormone [12]. Maximum plasma concentrations of GH are reached two to six hours after injection. The effects of GH are focused on minimizing protein loss through oxidation and increasing body and muscle mass [13]. Furthermore, GH plays an important paracrine effect on the central nervous system [14], where thyroid-stimulating hormone (TSH) is also implicated in the normal development of the nervous system, too [14,15,16,17,18]. The role of GH in the adult nervous system is controversial, but it has been reported to exert neuroprotective and neuroreparative functions [7,8]. Accordingly, in the context of SCI, GH treatment combined with physical therapy might provide neuroprotection and prevent further loss of nerve tissue.

The last few years have witnessed substantial improvements in mass spectrometry (MS)-based proteomics techniques, including sample preparation, liquid chromatography MS hardware, and data analysis [19,20,21,22]. In the present study, we applied several proteomics strategies for the unbiased, non-targeted study of patients with chronic SCI treated with rehabilitation and with or without somatropin in an attempt to decode the role of rehabilitation and somatropin treatment in chronic SCI individuals. Furthermore, we try to identify recovery markers in blood plasma samples in these kinds of patients.

## 2. Materials and Methods

### 2.1. Patient Recruitment and Study Design

Patient selection and classification was carried out by Physical Medicine and Rehabilitation department of the Hospital Nacional de Parapléjicos (Toledo, Spain). Forty-six patients with chronic SCI classified by lesion level (C4 and T12) were followed for a minimum period of one year every 6 months at the Neurology and Rehabilitation Department. The study was conducted according to the recommendations of the Declaration of Helsinki and was approved by the local Ethics Committee. In all cases (The ethic approval code is: 70/2010), informed consent was requested from all subjects participating in the study.

**Figure 1:**
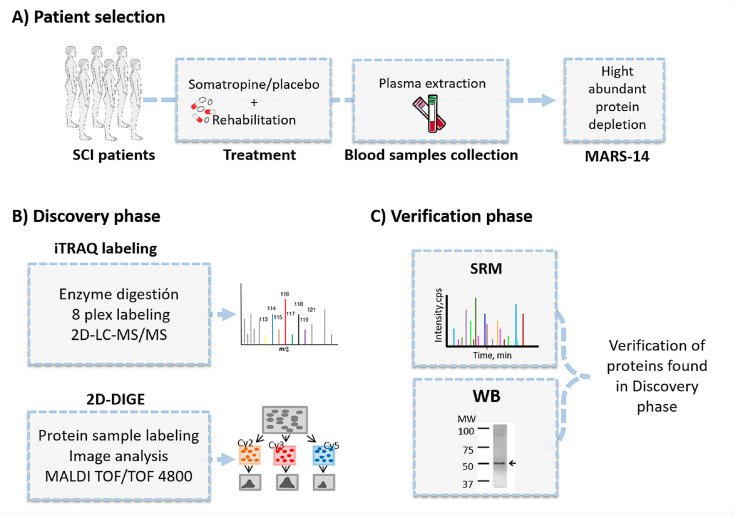
Experimental workflow. Stage 1 (**A**): Patient selection; fourteen high-abundance proteins were immunodepleted in plasma samples after extraction. Stage 2 (**B**): Depleted plasma samples were used in a discovery phase using two complementary proteomics techniques: isobaric tags for relative and absolute quantitation and two-dimensional fluorescence difference gel electrophoresis. Stage 3 (**C**): An independent cohort of patients was used in the verification phase using selected reaction monitoring.

After considering the inclusion and exclusion criteria (Table 1), patients were randomly divided into two groups (treatment and control). Randomization was achieved using a computer randomization program, and the records were held in the hospital Neurology Department (centralized randomization). A subgroup of the clinical trial also participates to this study. Overall, 23 patients received a daily dose of somatropin (recombinant somatropin–Genotonorm Miniquick^®^, 0.4 mg) over 364 days and also rehabilitation during the first six months. Twenty-three patients received placebo injection and rehabilitation following the same protocol (Figure 1). The proteomic study comprised two phases, a discovery phase and a validation phase. For the discovery phase, 26 patients with SCI were classified depending on pharmacological treatment: recombinant somatropin (group A) or placebo (group B). The discovery phase was followed by a verification phase, where we examined the proteins of interest in an independent cohort of patients (*n* = 20) with the objective of validating our findings in a different SCI population. All patients were matched by baseline characteristics and medication (Table 2).

For the proteomics analysis, 7 mL of blood was drawn into ethylenediaminetetraacetic acid (EDTA)-prepared collection tubes (Venoject, Terumo Europe, Leuven, Belgium). Samples were immediately taken to our laboratory (within 2 h) to minimize sample degradation. Samples were centrifuged at 3500× *g* for 10 min at 4 °C, and the resulting plasma was aliquoted in batches of 500 μL and stored at −80 °C until proteomic analysis. Plasma samples were collected at baseline and at 6 months. Patients were followed up for 1 year after the start of the study.

### 2.2. Motor Score Evolution and Measurement of Insulin-Like Growth Factor-1 Plasma Levels

Motor score evaluation was performed for each patient at baseline and at 6 months. Measurement of IGF-1 was performed at the hospital Clinical Analysis Laboratory using commercial ELISA kits (Thermo Fisher Scientific, Watham, MA, USA).

### 2.3. Proteomics Overview

The experimental strategy consisted of the following: (1) immunodepletion of the 14 more abundant plasma proteins [23]; (2) a discovery phase using two different, complementary, and robust proteomics techniques: two-dimensional fluorescence difference gel electrophoresis (2D-DIGE; GE Healthcare, Piscataway, NJ, USA) (*n* = 18) and isobaric tags for relative and absolute quantitation (iTRAQ) labeling (AB Sciex, Framingham, MA, USA) followed by liquid chromatography-tandem MS (*n* = 16) [24]; and (3) a verification phase in an independent cohort of 20 patients by two different orthogonal techniques: Western blotting and selected reaction monitoring (SRM) [25] (Figure 1).

#### 2.3.1. Sample Preparation for Proteomic Analyses

Immunodepletion was performed as described [23] using a system based on a high-performance liquid chromatography (affinity) column (HuMARS14; Agilent Technologies, Palo Alto, CA, USA) able to retain 14 abundant plasma proteins, thereby enhancing the detection and identification of low abundance proteins. Then, we performed a buffer exchange, as the elution buffer used in the immunodepletion has a high salt concentration that interferes with isoelectric focusing (IEF) and the iTRAQ protocol. Buffer exchange into Tris 20 mM, 2 M urea, 0.5 mM phenylmethylsulfonyl fluoride (PMSF) and 0.5 mM EDTA was performed using 3-kDa cut-off spin concentrators (Amicon Inc., Beverly, MA, USA) by centrifugation for 35 min at 3000× *g*.

#### 2.3.2. iTRAQ

Eight biological replicates for each treatment arm, each composed from four individuals, were independently analyzed in two 8-plex iTRAQ (Thermo Fisher Scientific Waltham, MA, USA) labeling experiments. A total of 100 μg of protein from each pool was cleaned by acetone precipitation, and the pellet was dissolved in 50 mM Tris, 4% SDS, 50 mM dithiothreitol (DTT; Sigma-Aldrich, Madrid, Spain) adjusted to pH 8 and then loaded onto SDS-PAGE gels to concentrate the proteins in a single band. Proteins were digested using an in-gel digestion protocol as described [26], with some modifications. Briefly, the supernatants were run by conventional SDS-PAGE until the front entered 3 mm into the resolving gel. The protein band containing the whole proteome was visualized by Coomassie staining, excised, cut into cubes, subjected to reduction with 10 mM DTT and alkylation in 50 mM iodoacetamide, and digested overnight at 37 °C with 60 ng/mL modified trypsin (Promega, Madison, WI, USA) at a 12:1 protein:trypsin (*w*/*w*) ratio in 50 mM ammonium bicarbonate, pH 8.8 containing 10% acetonitrile. The resulting tryptic peptides were extracted by incubation in 12 mM ammonium bicarbonate pH 8.8 and later, in 0.5% Trifluoroacetic acid (TFA). TFA was added to a final concentration of 1%, and the peptides were finally desalted onto C18 Oasis-HLB cartridges (Waters Corp., Medfor, MA, USA) and dried-down for further analysis.

##### Identification by LC-MS/MS

For stable isobaric labeling, the tryptic peptides were dissolved in triethylammonium bicarbonate buffer, and the concentration of peptides was determined by measuring amide bonds with the Direct Detect System (Millipore, Darmstadt, Germany). Equal amounts of each peptide sample were labeled using the 8-plex iTRAQ Reagents Multiplex Kit (Applied Biosystems, Foster City, CA, USA) according to the manufacturer’s protocol.

iTRAQ-labeled peptides were loaded onto an LC-MS/MS system for on-line desalting onto C18 cartridges and then analysis using a C-18 reversed-phase nano-column (75 μm I.D. × 50 cm, 2 µm particle size, Acclaim PepMap RSLC, 100 C18; Thermo Fisher Scientific) with a continuous acetonitrile gradient consisting of 0–30% B in 360 min, 50–90% B in 3 min (A = 0.5% formic acid; B = 90% acetonitrile, 0.5% formic acid). A flow rate of 200 nL/min was used to elute peptides from the nano-column to an emitter nanospray needle for real-time ionization and peptide fragmentation on a Q-Exactive mass spectrometer (Thermo Fisher). An enhanced fourier Transform (FT)-resolution spectrum (resolution  =  70,000) followed by the MS/MS spectra from the 15 most intense parent ions were analyzed along the chromatographic run. Dynamic exclusion was set at 30 s. To increase the proteome coverage, iTRAQ-labeled samples were also fractionated by cation exchange chromatography (Oasis HLB-MCX columns) into six fractions, which were desalted and analyzed using the same system and conditions described above.

##### iTRAQ Data Analysis

For peptide identification, all spectra were analyzed with Proteome Discoverer (version 1.4.0.29) using SEQUEST-HT (both from Thermo Fisher Scientific). To perform database searching at the Uniprot database containing all sequences from human and contaminants (25 March 2018), the following parameters were selected: trypsin digestion with 2 maximum missed cleavage sites, precursor and fragment mass tolerances of 2 Da and 0.02 Da, respectively, carbamidomethyl cysteine as fixed modification, and methionine oxidation as dynamic modifications. For iTRAQ-labeled peptides, N-terminal and Lys iTRAQ modifications were selected as a fixed modification. Peptide identification was validated using the probability ratio method [27] with an additional filtering for precursor mass tolerance of 12 ppm. The false discovery rate (FDR) was calculated using inverted databases and the refined method [28] with an additional filtering for precursor mass tolerance of 10 ppm. Only peptides with a confidence of at least 95% were used to quantify the relative abundance of each peptide determined, as described [26]. Protein quantification from reporter ion intensities and statistical analysis of quantitative data were performed using QuiXoT, based on a statistical model [29]. In this model, protein log2-ratios are expressed in form of the standardized variables; that is, in units of standard deviation according to their estimated variances (Zq values).

### 2.4. Two-Dimensional Differential Gel Electrophoresis

Prior to 2-DIGE analysis, samples were precipitated and cleaned using the Clean-Up commercial kit (GE Healthcare). The pellet was resuspended in labeling buffer (7 M urea 7 M, 2 M thiourea, 4% CHAPS and 30 mM Tris). Then, protein concentration was quantified using the Bradford–Lowry method [30].

#### 2.4.1. Sample Labeling

We labeled the proteins from the depleted plasma samples using the CyDye DIGE Fluor Minimal Labeling Kit (GE Healthcare). Briefly, 50 μg of each pool was labeled alternatively with 400 pmol of either Cy3 or Cy5 fluorescent dyes, and Cy2 was used to label a pooled internal standard with a mixture of all analyzed samples (pool of all 18 plasma samples). This internal standard was used for all 9 DIGE gels for comparison. After 30 min of incubation on ice in the dark, we quenched the reaction with 10 mM lysine incubation for 10 min.

#### 2.4.2. Two-Dimensional Electrophoresis

Labeled samples were applied by passive rehydration (rehydration buffer: 7 M urea 7 M, 2 M thiourea, 4% 3-[(3-Cholamidopropyl)dimethylammonio]-1-propanesulfonate hydrate (CHAPS), 40 mM DTT and ampholytes) overnight in immobilized pH gradient strips (IPG; 24 cm), pH 4–7 (Bio-Rad Laboratories, Hercules, CA), and subjected to isoelectric focusing on an IPGphor III unit (GE Healthcare, Chicago, IL, USA) using the following program: 30 min at 500 V, 3 h at 3500 V (gradient), 3 h step and hold at 3500 V, 3 h at 6000 V (gradient), and 6000 V for a total of 69,000 V/h. Then, strips were subjected to two successive incubations in an equilibration buffer (1.5 M Tris-HCl pH 8.8, 6 M urea, 87% *v*/*v* glycerol, and 2% *w*/*v* SDS). In the first incubation, we added DTT (1% [*w*/*v*]) to reduce the thiol groups of denatured proteins; in the second equilibration step, iodoacetamide (2.5% [*w*/*v*]) was added to alkylate reduced groups to avoid protein reoxidation.

#### 2.4.3. Image Acquisition and Analysis

The second dimension was carried out on 10% running gels, and fluorescence images of the gels were acquired on a Typhoon 9400 scanner (GE Healthcare, Chicago, IL, USA) using appropriate individual excitation and emission wavelengths, filters, and photomultiplier (PTM) values that are sensitive for each of the Cy3, Cy5, and Cy2 dyes (PTM values: 480, 490, and 500 nm, respectively).

Relative protein quantification was performed on AS and healthy valves with DeCyder software v6.5 (GE Healthcare, Chicago, IL, USA) and the multivariate statistical module EDA (Extended data analysis). The Differential in-gel analysis (DIA) module co-detected the 3 images of a gel (the internal standard and the two samples), measured the spot abundance in each image, and expressed these values as Cy3/Cy2 and Cy5/Cy2 ratios.

Then, these DIA datasets were analyzed using the Biological Variation Analysis module (BVA), which enabled the spot maps to be matched and the Cy3/Cy2 and Cy5/Cy2 ratios to be compared. Only protein spots with >1.3-fold differences in abundance were considered for the analysis. Then, a statistical analysis was carried out to determine the changes in protein species, with *p*-values below 0.05 accepted as significant.

Finally, a multivariate analysis was performed by Principal Component Analysis (PCA) using the algorithm included in the EDA module of the DeCyder software (version 6.5) based on the spots that matched across all the gels. The differential in-gel analysis module allowed for the co-detection and quantification of spots of the three fluorescent images of each gel. The biological variation analysis module enabled the differential abundance analysis of matched spots. Statistical analysis was performed, and *p*-values ≤ 0.05 and fold-change ≥ 1.3 were considered of interest. A pattern analysis hierarchical classification was obtained using the Pearson coefficient based on the spots present in 90% of all the gels.

Then, the gels were re-stained with a silver staining kit (GE-Healthcare, Chicago, IL, USA), as described previously [31].

#### 2.4.4. Protein Identification by Mass Spectrometry

Differentially expressed protein spots were either excised directly from the re-stained DIGE gels or from preparative gels using 400 μg of total protein, following the same procedures as described for the DIGE gels. The excised spots were digested at 37 °C using a published protocol [32] with minor variations gel plugs that were subjected to reduction with 10 mM dithiothreitol (Sigma Aldrich) in 50 mM ammonium bicarbonate (99% purity; Scharlau) and alkylation with 55 mM iodoacetamide (Sigma Aldrich) in 50 mM ammonium bicarbonate. Then, the gel pieces were rinsed with 50 mM ammonium bicarbonate in 50% methanol (gradient, HPLC grade, Scharlau) and acetonitrile (gradient, HPLC grade, Scharlau) and dried in a Speedvac. Modified porcine trypsin (sequencing grade; Promega, Madison, WI, USA) at a final concentration of 20 ng/µL in 20 mM ammonium bicarbonate was added to the dry gel pieces, and the digestion proceeded at 37 °C overnight. Finally, 70% aqueous acetonitrile and 0.1% formic acid (99.5% purity; Sigma Aldrich) was added for peptide extraction.

After overnight digestion at 37 °C, the peptides were extracted with 60% acetonitrile (ACN) in 0.1% formic acid (99.5% purity; Sigma Aldrich). Then, the samples were dried in a Speedvac and resuspended in 98% water with 0.1% formic acid (FA) and 2% ACN. An aliquot of each digestion was mixed with an aliquot of the matrix solution (3 mg/mL α-cyano-4-hydroxycinnamic acid: Sigma Aldrich) in 30% ACN, 15% 2-propanol, and 0.1% TFA, and this mixture was pipetted directly onto the stainless steel sample plate of the mass spectrometer 384 Opti-TOF 123 × 81 mm MALDI (Applied Biosystems) and dried at room temperature.

The MALDI-MS/MS data were obtained in an automated analysis loop using a 4800 Plus MALDI TOF/TOF Analyzer (Applied Biosystems). Spectra were acquired in the reflector positive-ion mode with a Nd:YAG, 355 nm wavelength laser at a frequency of 200 Hz, and between 1000 and 2000 individual spectra were averaged. The experiments were acquired in a uniform mode with fixed laser intensity. For the MS/MS 1 kV analysis mode, precursors were accelerated to 8 kV in source 1, and they were selected at a relative resolution of 350 Full Width at Half Maximum (FWHM) and with metastable suppression. Fragment ions generated by collision with air in a Collision-induced dissociation CID chamber were further accelerated at 15 kV in source 2. Mass data were analyzed automatically with the 4000 Series Explorer Software version 3.5.3 (Applied Biosystems Foster City, CA, USA). Internal calibration of MALDI-TOF mass spectra was performed using two trypsin autolysis ions with m/z = 842.510 and m/z = 2211.105. For the calibrations in the MS/MS mode, the fragment ion spectra obtained from Glub-fibrinopeptide were used (4700 Cal Mix, Applied Biosystems). MALDI-MS and MS/MS data were combined through the GPS Explorer Software (Version 3.6) to search a non-redundant protein database (Swissprot 56.5) using the Mascot software (version 2.2: Matrix Science Inc., Boston, MA, USA) [33]. The following parameters were employed: 50 ppm precursor tolerance; 0.6 Da MS/MS fragment tolerance; 1 missed cleavage permitted; and with carbamidomethyl cysteines and methionine oxidation as a modification. MALDI-MS (/MS) spectra and database search results were manually inspected using the aforementioned software. For combined MS and MS/MS data, identifications were accepted when Confidence Interval (C.I. %) of GPS software was 95% or higher. Since Protein Scores and Ion Scores from different searches cannot be directly compared, GPS software calculates this C.I. % in order to combine results from MS and MS/MS database searches. This coefficient value means that the probability that the observed match is a random event is lower than 5%. For PMF spectra, identifications were also accepted when (C.I. %) of GPS software was 99% or higher. Alternatively, the ppw files were obtained from 4000 series explorer software (ABSciex, Madrid, Spain). Peptides were analyzed in a 4800 Plus MALDI TOF/TOF (AB Sciex, Madrid, Spain) mass spectrometer, and protein identifications were achieved by subjecting the obtained MS/MS spectra to the MASCOT 2.2 (Matrix Science Inc., Boston, MA, USA) search engine, using the Swissprot 56.2 database with 50 ppm precursor tolerance, 0.6 Da MS/MS fragment tolerance, carbamidomethyl cysteine as fixed modification, oxidized methionine as variable modification, and allowing 1 missed cleavage.

### 2.5. Western Blotting

Twenty samples from an independent cohort of patients were used for Western blot analysis—10 patients were treated with somatropin and 10 were treated with placebo, at baseline and at 6 months. Proteins (25 μg) were separated by 12% SDS-PAGE and analysis was carried out using anti-vitamin D binding protein (GC) (1/1000), anti-plasminogen (PLG) (1/2000), antibodies, and species-specific secondary antibodies, all from Abcam (Cambridge, UK). Relative protein levels were determined by densitometry using ImageQuant software (Amersham, Uppsala, Sweden).

### 2.6. Selected Reaction Monitoring

Following our previously published protocol [34,35], samples were reduced with 100 nM DTT in 50 mM ammonium bicarbonate (99% purity; Scharlau, Barcelona, Spain) for 30 min at 37 °C and alkylated with 550 mM iodoacetate in 50 mM ammonium bicarbonate for 20 min at room temperature. Proteins were digested in 50 mM ammonium bicarbonate, 15% acetonitrile (LC-MS grade; Scharlau, Barcelona, Spain) with sequencing-grade modified porcine trypsin (Promega, Madison, WI, USA) at a final concentration of 1:50. After digestion at 37 °C overnight, 2% formic acid (99.5% purity; Sigma-Aldrich) was added, and samples were cleaned using Pep-Clean spin columns (Pierce, Rockville, MD, USA). Tryptic digests were dried in a Speed Vac and resuspended in 2% acetonitrile, 2% formic acid prior to MS analysis. The LC-MS/MS system consisted of a TEMPO nano LC system (Applied Biosystems) combined with a nano LC autosampler and coupled to a modified triple quadrupole MS system (Applied Biosystems 4000 QTRAO LC/MS/MS). Three replicate injections (4 μL containing 20 μg of protein) were performed per sample using mobile phase A (2% acetonitrile/98% water, 0.1% formic acid) with a flow rate of 10 μL/min for 5 min. Peptides were loaded onto a μ-Precolumn Cartridge (Acclaim Pep Map 100 C18, 5 μm, 100 Å; 300 μm I.D. × 5 mm; LC Packings, Idstein, Germany) to pre-concentrate and desalt samples. Reverse phase LC was achieved on a C18 column (Onyx Monolithic C18, 150 × 0.1 mm I.D.; Phenomenex, Torrance, CA, USA) in a gradient of phase A and phase B (98% acetonitrile/2% water, 0.1% formic acid). Peptides were eluted at a flow rate of 900 nL/min in the following steps: 2–15% B for 2 min, 15–30% B for 18 min, 30–50% B for 5 min, 50–90% B for 2 min, and finally 90% B for 3 min. Then, the column was regenerated with 2% B for 15 additional minutes. Both the 2-(2,2,6,6-tetramethyl piperidine-1-oxyl (TEMPO) nano LC and 4000 QTRAP system were controlled by Analyst Software v.1.4.5. The mass spectrometer was set to operate in positive ion mode with an ion spray voltage of 2800 V and a nanoflow interface heater temperature of 150 °C. Source gas 1 and curtain gas were set to 20 and 20 psi, respectively, and nitrogen was applied as both curtain and collision gases. Collision energy was optimized to obtain maximum transmission efficiency and sensitivity for each SRM transition. Two or three transitions per peptide were monitored during an individual sample analysis. They were acquired at unit resolution in both Q1 and Q3, with dwell times from 40 to 120 ms, resulting in cycle times of 4.0957 s. The IntelliQuan algorithm, included in Analyst 1.4.5 software, was used to calculate abundances based on peak areas after integration. To calculate significant differentially abundant proteins, we first calculated the concentration for each peptide according the following equation (fmoL/H *50) corresponding to 2 µg of digested sample. Secondly, we calculated an average of all identified peptides. Significance was considered with a *p*-value ≤ 0.05 (Table 5).

### 2.7. Statistical Analysis

Statistical analyses were performed using SPSS 15.0 for windows software (SPSS Inc., Chicago, IL, USA). Nonparametric analyses were performed to analyze data. The Wilcoxon test was used in case of related samples and the Mann–Whitney U test was used in case of independent samples. Results were expressed as means ± standard deviation (SD). For iTRAQ analysis, we considered the differentially expressed proteins identified with at least 1 peptide and log2-ratios expressed in the form of the standardized variables (Zq ± 1.5) with *p*-values ≤ 0.05, with Zq being the mean for the 2 replicates versus the placebo group. In relation to the 2D-DIGE analysis, a fold change of 1.3 was imposed and *p* ≤ 0.05 was considered as significant differences. For SRM analysis, we considered the mean of all the peptides separately used to identify the protein in each case. Significance was considered with a *p*-value ≤ 0.05.

Pearson’s correlation coefficient was calculated to analyze the association between two variables. In groups treated with somatropin, several correlations were performed—motor score and IGF-1, and motor score with GC—both at 6 months of treatment. These correlations could help us better understand the grouping of the subjects after PCA analysis. Significance was accepted at *p* ≤ 0.05 in all cases.

## 3. Results

We sought to evaluate whether protein alterations could identify those SCI individuals who will respond to somatropin or reahabilitation treatment. We performed quantitative proteomics analyses in a transversal study using plasma samples collected at baseline (collected before treatment) and at 6 months. Table 1 describes patients’ clinical characteristics. No significant differences between the treatment and placebo groups were found for sex and age. Likewise, motor score parameters and IGF-1 levels were not significantly different between groups (at baseline and at 6 months).

**Figure 2:**
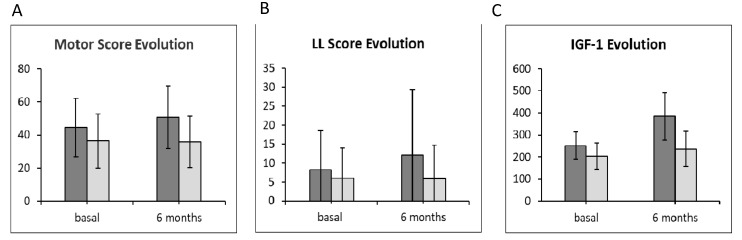
(**A**) Mean per group of motor score changes at different time points: basal and 6 months. (**B**) Mean per group of lower limb (LL) score changes at different times: basal and 6 months (**C**) Evolution of insulin growth factor-1 (IGF-1) in each study group at different times: basal and 6 months. Dark gray bar: growth hormone (GH) group; light gray bar: placebo group.

We re-measured some clinical parameters after 6 months to test for improvements in patients who received somatropin or placebo. Regarding the evolution of motor score, we observed a relatively slight improvement in patients treated with somatropin (Figure 2A). These patients showed an increase both in total motor score (Figure 2A) and lower limb score (Figure 2B), whereas no significant improvements were observed in patients treated with placebo (Figure 2A,B). Analysis of IGF-1 levels at 6 months revealed an increase in the somatropin treatment group but not in the placebo group (Figure 2C).

**Figure 3:**
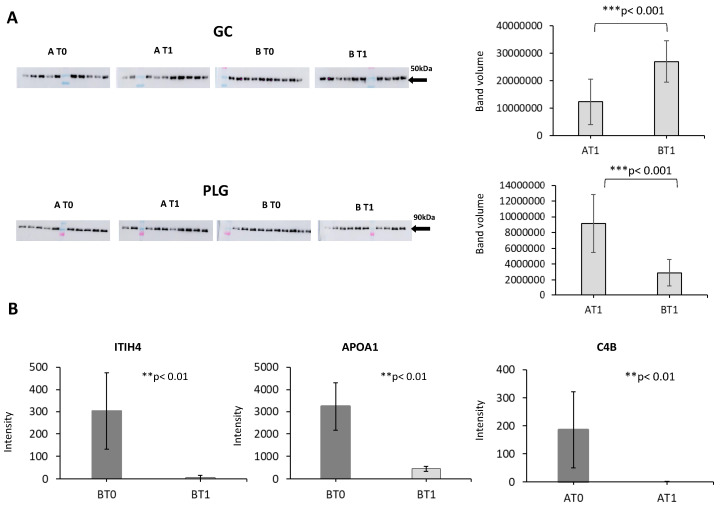
(**A**) Verification of GC and anti-plasminogen (PLG) by Western blotting. Volume represents band intensity band determined by Image Quant QL software: AT0 (group treated with somatropin, at baseline), AT1 (group treated with somatropin at 6 month), BT0 (group treated with placebo, at baseline), and BT1 (group treated with placebo at 6 months). (**B**) Verification of ITIH4, APOAI, and C4B by selected reaction monitoring. AT0 (group treated with GH, at baseline time). AT1 (group treated with GH at 6 months), BT0 (group treated with placebo, at baseline), BT1 (group treated with placebo at 6 months).

We searched for molecular changes occurring in the plasma proteome of patients during somatropin treatment using iTRAQ and 2D-DIGE proteomic approaches. The iTRAQ liquid chromatography-tandem mass spectrometry strategy allowed us to identify 984 proteins in total, of which 21 were differentially expressed (Table 3). The 2D-DIGE analysis revealed 11 protein spots differentially expressed between the two groups, which correspond to eight proteins (Table 4). Of special note, the eight proteins were not detected as significantly expressed by iTRAQ, so the combination of these two different and complementary proteomics techniques guarantees a more extensive coverage of quantified proteins [36]. To validate these changes, and considering the fold-change and the possible involvement of molecular mechanisms related to SCI pathology, we analyzed 10 proteins by SRM and five by Western blotting in an independent cohort of 20 patients with a good response in the motor score. Two of the five proteins were verified by Western blotting (PLG, GC) (Figure 3A), and three of the 10 proteins were verified by SRM (APOA1, C4B, and ITIH4) (Figure 3B).

Non-parametric test (Mann–Whitney U test) of PLG (≈90 kDa) and GC (50 kDa) revealed significant differences between the somatropin and placebo groups at 6 months after the start of treatment (*p* < 0.001 and *p* < 0.001, respectively), with the levels of PLG increased in the somatropin-treated group and the levels of GC decreased. Of the three proteins validated by SRM (Figure 3B) (Table 5), the data obtained corroborated the proteomics results. ITIH4 and APOA1 were downregulated in the placebo group after 6 months, and C4B was likewise downregulated in the somatropin group.

**Figure 4:**
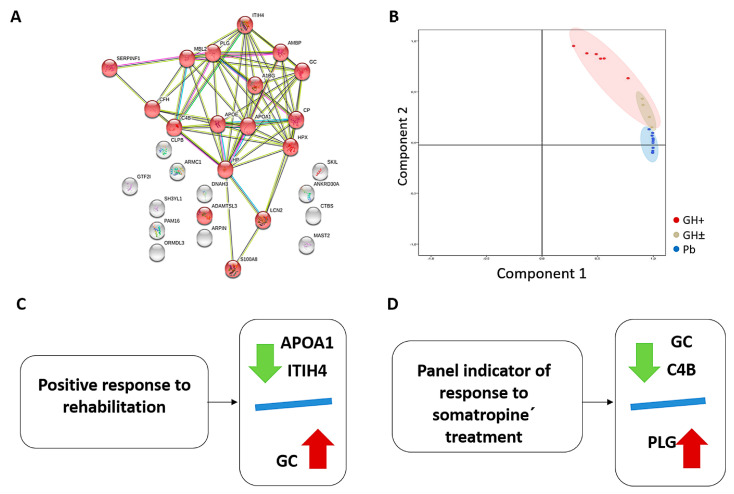
(**A**) Pathway analysis of the differentially expressed proteins. Protein–protein interaction networks were studied with STRING v11.0 software. Proteins with red circles correspond to proteins that are implicated on the extracellular region. (**B**) Principal Component Analysis with the validated proteins showed the correct grouping of patients treated with placebo (Pb). Patients treated with somatropin formed two sub-groups: a positive response (GH+) and a non-response (GH±) to somatropin. (**C**) Panel of response to rehabilitation which is composed of three proteins (APOA1, ITIH4, GC) showing downregulation in the placebo group at 6 months. (**D**) Panel indicator of response to somatropin treatment: GC and C4B were downregulated, whereas PLG was upregulated at 6 months.

The biological implication of the differentially expressed proteins detected both by 2D-DIGE analysis and by iTRAQ analysis were analyzed using DAVID Bioinformatics Resources 6.7 software. Interestingly, the validated proteins that belonged to one cluster that corresponded to the extracellular region showed an enrichment score = 7.11, and *p* = 2.9 × 10^−10^. Functional analysis of the differentially expressed proteins was explored using the String v11.0 webtool (Figure 4A). According to the molecular function and the pathway analysis performed, it is important to note that proteins validated by Western blotting and SRM corresponded to the extracellular region and showed a significant interaction with each other with a false discovery rate = 4.82 × 10^−07^ (Figure 4A).

We also performed PCA with the validated proteins (Figure 4B), which showed the correct grouping of patients treated with placebo. By contrast, patients treated with somatropin formed two sub-groups: a positive response (GH+) and a non-response (GH±) to somatropin. These results reveal that patients with no response to somatropin are similar to patients treated with placebo.

According to the PCA, the five proteins validated by Western blotting and SRM analysis were divided into two panels: (i) indicator of response to somatropin treatment: GC, C4B, and PLG (Figure 4C) and (ii) positive response to rehabilitation (this went down well in the placebo group): GC, APOA1, and ITIH4 (Figure 4D).

**Figure 5:**
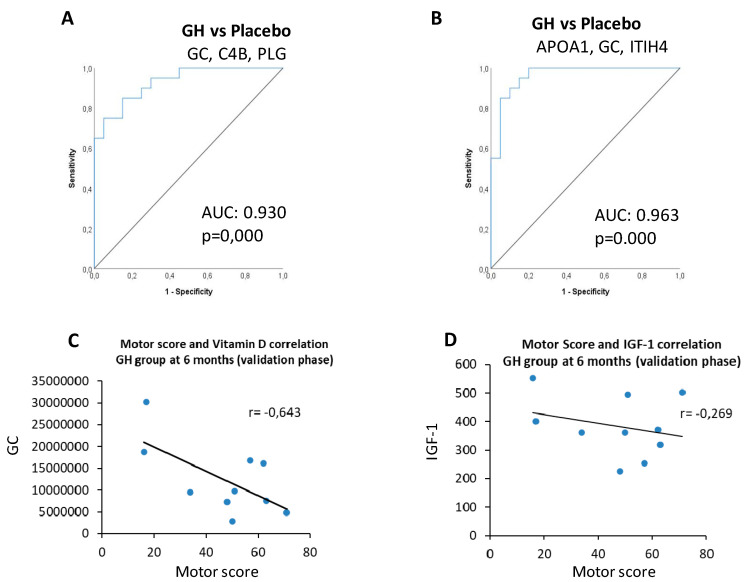
(**A**) Receiver operating characteristic (ROC) curve of the panel indicator of response to somatropin formed by three proteins. Area under the ROC curve (AUC) and *p*-values are shown. (**B**) ROC curve of the rehabilitation panel formed by three proteins. AUC and *p*-value are shown. (**C**) Negative correlation between somatropin and motor score. (**D**) Negative correlation between IGF-1 and motor score.

Receiver operating characteristic (ROC) curve analysis showed a good sensitivity and specificity for both panels. The indicator of response to somatropin panel could discriminate somatropin from placebo treatment with an area under the ROC curve (AUC) of 0.930 (Figure 5A). The indicator of positive response to rehabilitation panel had an AUC of 0.963 (Figure 5B).

To search for links between clinical parameters and the validated proteins, we performed correlation analyses of the proteins validated and motor score levels. We found that GC had a negative correlation with motor score levels in patients at 6 months of treatment (r = −0.643) (Figure 5C). In addition, we found a negative correlation between IGF-1 and motor score (r = −0.269) (Figure 5D). These results indicate that higher levels of IGF-1 and GC imply a worse somatropin treatment response.

## 4. Discussion

No curative treatment yet exists for SCI, and rehabilitation is the only therapeutic option that can contribute to functional improvement in patients. Mass spectrometry proteomics has become a powerful tool in biomedical research over the past 20 years [37], including its applications for studying the differential proteome in SCI [38,39]. In this context, here, we utilized an unbiased proteomic approach with two independent but complementary techniques (iTRAQ and 2D-DIGE) to search for protein biomarkers as indicators of improvement associated with the administration of GH in patients with SCI. These approaches revealed 21 and eight differentially expressed proteins, respectively, with GH treatment, and five of these protein biomarkers were confirmed by SRM and Western blotting in an independent cohort of 20 patients. These evident differential changes to the SCI proteome allow us to molecularly characterize several processes implicated in GH treatment and associated with SCI.

All six proteins are related to the extracellular region (the space external to the outermost structure of a cell), pointing to the activation of this pathway in patients with SCI treated with GH. Moreover, ROC curves showed good sensitivity and specificity for these protein markers, pointing to the potential value of these indicators to classify patients with SCI according to treatment. A panel of three proteins (C4B, GC, and PLG) was altered in the plasma from patients treated with somatropin and might have a potential predictive value in estimating the improvement of SCI after somatropin treatment, which is also supported by the ROC curve value (AUC = 0.930) (Figure 5A) and by PCA analysis (Figure 4B).

We found a different proteomic signature made up of three proteins (APOA1, GC, and ITIH4) that are differentially expressed in the placebo group, and whose levels might be related to positive response to rehabilitation. Again, this result is supported by the ROC curve analysis (AUC = 0.963) (Figure 5B).

### 4.1. Panel Indicator of Response to Somatropin Treatment

The complement system, which includes C4B, plays a critical role in development, homeostasis, and regeneration in the central nervous system (CNS) throughout life [40]. The complement cascade is at the interface of the coagulation and inflammation pathways and is operative in acute neurological disorders, such as SCI and traumatic brain injury [41]. This is in line with our proteomics data, which showed an upregulation of C4B along the time in the treated group.

LG is a glycoprotein that circulates in plasma as a zymogen and, when converted to proteolytically active plasmin, dissolves preformed fibrin clots and extracellular matrix components [42]. A regulatory role for PLG has been described in complement and coagulation cascades [43], and it is also implicated in inflammation pathways that occur in SCI [40]. Those of the IGF-1 protein include controlling the regeneration of injured peripheral nerves, reducing the progression of cell death, inducing progenitor cell differentiation, and promoting neurite outgrowth [44,45]. Furthermore, it has been described that PLG activators play a functional role in synaptic plasticity associated with the crossed phrenic phenomenon and recovery of respiratory function following SCI [46]. We found that IGF-1 levels increased in patients treated with somatropin. This might be due to the action of somatropin, which could be reflected in the higher PLG levels.

It is important to note that bone loss occurs rapidly during the first 4–6 months to one year post SCI, with a 40% reduction in sub-lesional bone mineral density reported at two years post injury [47,48]. It has been described that osteoporosis following SCI results from the deficiency of GC and by the pituitary suppression of TSH [49,50]. This is in accord with our results, as we found a decrease in plasma GC at 6 months in patients treated with somatropin. This is also corroborated by our findings of a negative correlation between motor score and GC level, with higher levels of vitamin D binding protein implying a worse response to somatropin treatment.

### 4.2. Panel Indicator of Positive Response to Rehabilitation

APOA1 and ITHIH4 abundance were reduced in the placebo group over time. Nevertheless, the patients in this group showed an improvement in their physical condition as assessed by their motor score values, which is likely due to the rehabilitation that they received along the study. ITIH4 is a serine protease inhibitor involved in extracellular matrix stabilization and in inflammatory response [51]. ITIH4 binds hyaluronan, which is an integral component of the CNS extracellular matrix and modulates the level of free hyaluronan [52]. In the acute phase of SCI, the CNS is damaged and the accumulation of free hyaluronan contributed to the inhibition of re-myelination and axonal regeneration, as well as the onset of inflammation [53]. As shown by our results, inflammation and extracellular matrix components may be stabilized and improved during the rehabilitation therapy, and therefore, ITIH4 levels decrease.

APOA1 is believed to control inflammatory activities after CNS injury and it ameliorates the tissue damage that accompanies uncontrolled inflammation, and it also promotes axonal repair [54]. Our results are similar to those in the literature, as in the placebo group, APOA1 was higher at baseline than after 6 months. The inflammation phase of SCI occurs during the first months of injury, in which APOA1 is described to be downregulated [55].

In this study, we highlight the potential of plasma proteomics to discover novel indicators of response to somatropin treatment or response to rehabilitation in patients with SCI. Our study provides a subset of promising markers of response to somatropin treatment or response to rehabilitation, as suggested by the high AUC values. These panels could shed some light on the SCI regulatory mechanisms triggered in response to somatropin treatment and rehabilitation. Indeed, the proteins described here are all implicated in inflammation, homeostasis, and coagulation processes, which are initial and secondary events of SCI. Furthermore, a remarkable feature of this study is the potential of rehabilitation to improve function in patients.

The major limitation to our study is the sample size, and larger samples will be needed to confirm the potential value of the protein indicators described here. Future efforts should be directed toward performing a functional analysis of these candidate markers to assess their potential role in improving function in patients with SCI. A prospective study will be required to determine the clinical utility of these proteins as potential biomarkers to distinguish those patients who will have a positive response to GH treatment and those who will be positive responders to rehabilitation without the need for GH treatment.

## Figures and Tables

**Table 1:** Inclusion and exclusion criteria.

Inclusion Criteria	Exclusion Criteria
Age between 18 and 75 years Incomplete SCI (AIS B or C) Etiology: traumatic Neurological level between C4 and T12 Time since injury > 18 months	Age < 18 or >75 years Etiology: Non-traumatic spinal cord injury Complete SCI (AIS A) Incomplete SCI (AIS D or E) Neurological level above C4 or below T12 Time since injury <18 months Intensive Care Unit stay for a period of ≥2 months More than 3 urinary tract infections in the last year Pneumonia in the 6 months prior to the study or severe respiratory failure History of head trauma Severe psychiatric disorder History of heart disease, diabetes or hypertension Concomitant neurological diseases Regular use of substances of abuse Severe kidney and/or liver failure. Impossibility to be included in an intensive rehabilitation program Pregnancy or breast-feeding History of malignancy Impossibility to obtain informed consent

**Table 2:** Clinical characteristics of subjects with spinal cord injury (SCI) and with combined rehabilitation and somatropin treatment. (CI, confidence interval).

Characteristics	Mean	GH Group	C I	Placebo Group	C I	*p*
**No of subjects**		**23**		23		
**Sex (Male)**		z				0786
Male	82.88					
Female	17.12					
**Age (y)**	35.00					0946
**AIS grade**	Only baseline					
A	Not include					NA
B	55.48					NA
C	44.42					NA
D	Not include					NA
**IGF-1**						
Baseline		195.83	28.50	199.66	27.48	05567
**Motor score**						
Baseline		44.44	6.99	36.38	6.95	0119

**Table 3:** Differentially expressed proteins identified by isobaric tags for relative and absolute quantitation (iTRAQ). Accession number (AccN), protein name and abbreviation (Abb.) according to Uniprot database. N: number of unique peptides. Zq: mean of log2-ratios expressed in form of the standardized variables for two replicates.

AccN	Protein Name	Abb	Functions	N	Zq Placebo Group	Zq GH Group	Power
P08603	Complement factor H	CFAH	Complement activation/Regulation of complement cascade	66	2.30	0.45	0.999
P00747	Plasminogen	PLMN	Blood coagulation/Catalytic activity/Extracellular matrix disassembly	39	2.19	0.33	0.999
P02760	Protein AMBP	AMBP	Metabolic process/Catalytic activity/Negative regulation of immune response	13	2.33	−0.01	1.000
P11226	Mannose-binding protein C	MBL2	Acute-phase response/Complement activation/Innate immunity	9	−0.56	−3.30	0.999
P00739	Haptoglobin-related protein	HPTR	Acute inflammatory response/ Positive regulation of cell death/Metabolic process	9	−2.58	6.27	0.999
Q01459	Di-N-acetylchitobiase	DIAC	Glycosidase/Hydrolase	3	−0.57	−1.92	0.999
P80188	Neutrophil gelatinase-associated lipocalin	NGAL	Innate immune response/Transport/Protease binding	2	1.67	−1.34	1.000
Q9Y3D7	Mitochondrial import inner membrane translocase subunit TIM16	TIM16	Ossification/Negative regulation of ATPase activity/Transport	2	−1.92	−0.31	0.995
D6RF35	Vitamin D-binding protein	D6RF35	Transport/Actin-binding	2	−1.58	1.19	1.000
Q8TD57	Dynein heavy chain 3, axonemal	DYH3	Cellular process/ATP binding/Microtubule-based movement	1	−1.39	3.73	1.000
Q9BXX3	Ankyrin repeat domain-containing protein 30A	AN30A	Regulation of transcription/DNA binding	1	−0.72	1.89	1.000
P12757	Ski-like protein	SKIL	Cell division and differentiation/Response to cytokine and growth factor/Positive regulation of axonogenesis	1	−0.72	1.75	0.999
Q7Z6K5	Arpin	ARPIN	Negative regulation of actin nucleation/Negative regulation of cell migration	1	−1.65	1.31	1.000
Q6P0Q8	Microtubule-associated serine/threonine-protein kinase 2	MAST2	Cytoskeleton organization/Intracellular signal transduction/Regulation of interleukin-12 biosynthetic process	1	2.40	−0.94	1.000
P78347	General transcription factor II-I	GTF2I	Negative regulation of angiogenesis/Transcription by RNA polymerase II/Signal transduction	1	−0.63	−2.52	1.000
P82987	ADAMTS-like protein 3	ATL3	Peptidase activity/Cellular process/Proteolysis	1	0.53	−10.78	0.950
Q9NVT9	Armadillo repeat-containing protein 1	ARMC1	Metal ion transport/Biological regulation/Metabolic process	1	1.63	−4.12	0.996
Q8N138	ORM1-like protein 3	ORML3	Cellular sphingolipid homeostasis/Ceramide metabolic process/Neutrophil degranulation	1	0.19	−2.17	1.000
Q96HL8	SH3 domain-containing YSC84-like protein 1	SH3YL1	Phosphatidylinositol biosynthetic process/Regulation of ruffle assembly	1	2.94	−0.01	0.999
P05109	Protein S100-A8	S10A8	Regulation of inflammatory processes and immune response/Positive regulation of cell growth/Astrocyte development/Wound healing	1	2.41	−0.70	0.999
H0YGM0	Caseinolytic peptidase B protein homolog	H0YGM0	Regulatory ATPase/Cellular response to heat/Protein binding	1	−0.31	−1.84	0.999

**Table 4:** Differentially expressed proteins identified by two-dimensional fluorescence difference gel electrophoresis (2D-DIGE). Differences in fold change are shown as average ratio (AvR). Protein name and accession number (AccN) according to Uniprot database are listed.

	AccN	Protein Name	Functions	Somatropin *t*0 vs. *t*1		Placebo *t*0 vs. *t*1	
				AvR	*p*-Value	AvR	*p*-Value
1	Q14624	Inter-alpha-trypsin inhibitor heavy chain H4	Serine-type endopeptidase inhibitor activity	1.55	7.70 × 10^−3^		
2	Q14624	Inter-alpha-trypsin inhibitor heavy chain H4	Serine-type endopeptidase inhibitor activity			−3.82	0.0106
3	P0C0L5	Complement C4-B	Inflammatory response/Complement activation	−1.24	0.0107		
4	P02647	Apolipoprotein AI	Cholesterol transport	1.65	0.0293		
5	P02647	Apolipoprotein AI	Cholesterol transport			−1.66	0.0474
6	P00450	Ceruloplasmin	Oxidoreductase	−1.15	0.0139		
7	P00450	Ceruloplasmin	Oxidoreductase			−1.5	0.0175
8	P36955	Pigment epithelium-derived factor	Serine-type endopeptidase inhibitor activity			−1.19	0.0472
9	P02649	Apolipoprotein E	Cholesterol metabolism			−1.11	0.0181
10	P02790	Hemopexin	Metalloprotease			−3.13	0.0332
11	P04217	Alpha-1B-glycoprotein	Neutrophil and platelet degranulation			−1.27	0.00545

**Table 5:** Monitored proteins by selected reaction monitoring (SRM) in plasma samples. Shown are measured peptides for each protein. Mean per peptide and mean per protein. Concentration expressed as fmol L/H * 50 corresponded to 2 µg of digested sample. Abb: protein abbreviation; AccN: accession number. APOA1: apolipoprotein A1; CO4B: complement C4-B; ITIH4: inter-alpha-trypsin inhibitor heavy chain H4.

AccN	Function	Abb	Peptide Sequence	Peptide Means	Protein Means	*p*-Value	
						Bt0 vs. Bt1	At0 vs. At1
Q14624	Protease inhibitor	ITIH4	ANTVQEATFQMELPK	595.12 ± 12.81	302.71 ± 6.66	**<0.001**	NA
			IPKPEASFSPR	10.30 ± 0.51			
P0C0L5	Complement activation; Inflammatory response	CO4B	VLSLAQEQVGGSPEK	31.62 ± 3.03	186.82 ± 1.65	NA	**<0.001**
			AEMADQAAAWLTR	342.03 ± 0.27			
P02647	Cholesterol transport	APOA1	DYVSQFEGSALGK	3234.20 ± 456.42	3234.20 ± 456.42	**<0.001**	NA
